# Sleep disturbance in people living with dementia or mild cognitive impairment: a realist review of general practice

**DOI:** 10.3399/BJGP.2023.0171

**Published:** 2024-03-19

**Authors:** Aidin Aryankhesal, Jessica Blake, Geoff Wong, Molly Megson, Simon Briscoe, Louise Allan, Niall M Broomfield, Zenahrai Eastwood, Leanne Greene, Andrea Hilton, Anne Killett, Alpar S Lazar, Rachael Litherland, Gill Livingston, Ian Maidment, Joanne Reeve, George Rook, Sion Scott, Jinpil Um, Jayden van Horik, Chris Fox

**Affiliations:** Faculty of Medicine and Health Sciences, University of East Anglia, Norwich.; Faculty of Medicine and Health Sciences, University of East Anglia, Norwich.; Nuffield Department of Primary Care Health Sciences, University of Oxford, Oxford.; Academy of Primary Care, Hull York Medical School, University of Hull, Hull.; University of Exeter Medical School, Exeter.; University of Exeter Medical School, Exeter.; Department of Clinical Psychology and Psychological Therapies, Norwich Medical School, University of East Anglia, Norwich.; Faculty of Medicine and Health Sciences, University of East Anglia, Norwich.; University of Exeter Medical School, Exeter.; School of Paramedical PeriOperative and Advanced Practice, Faculty of Health Sciences, University of Hull, Hull.; Faculty of Medicine and Health Sciences, University of East Anglia, Norwich.; Faculty of Medicine and Health Sciences, University of East Anglia, Norwich.; Innovations in Dementia, Exeter.; Faculty of Brain Sciences, Division of Psychiatry, University College London, London.; Aston Pharmacy School, College of Health and Life Sciences, Aston University, Birmingham.; Academy of Primary Care, Hull York Medical School, University of Hull, Hull.; The Lived Experience Advisory Panel.; School of Healthcare, College of Life Sciences, University of Leicester, Leicester.; University of Exeter Medical School, Exeter.; University of Exeter Medical School, Exeter.; University of Exeter Medical School, Exeter.

**Keywords:** caregivers, cognitive dysfunction, community health services, family practice, primary health care, sleep wake disorders

## Abstract

**Background:**

Sleep disturbance is a prevalent condition among people living with dementia (PLwD) or mild cognitive impairment (MCI). Its assessment and management within primary care is complex because of the comorbidities, older age, and cognitive impairment typical of this patient group.

**Aim:**

To explore how primary care clinicians assess, understand, and manage sleep disturbance for PLwD or MCI; if and why such initiatives work; and how people and their carers experience sleep disturbance and its treatment.

**Design and setting:**

A realist review of existing literature conducted in 2022.

**Method:**

Six bibliographic databases were searched. Context–mechanism–outcome configurations (CMOCs) were developed and refined.

**Results:**

In total, 60 records were included from 1869 retrieved hits and 19 CMOCs were developed. Low awareness of and confidence in the treatment of sleep disturbance among primary care clinicians and patients, combined with time and resource constraints, meant that identifying sleep disturbance was difficult and not prioritised. Medication was perceived by clinicians and patients as the primary management tool, resulting in inappropriate or long-term prescription. Rigid nursing routines in care homes were reportedly not conducive to good-quality sleep.

**Conclusion:**

In primary care, sleep disturbance among PLwD or MCI is not adequately addressed. Over-reliance on medication, underutilisation of non-pharmacological strategies, and inflexible care home routines were reported as a result of low confidence in sleep management and resource constraints. This does not constitute effective and person-centred care. Future work should consider ways to tailor the assessment and management of sleep disturbance to the needs of individuals and their informal carers without overstretching services.

## Introduction

Sleep disturbance can be defined as any condition that affects the quality, timing, or length of sleep so there is an impact on a person’s daily functioning.^1^ Sleep disturbance includes but is not limited to insomnia, narcolepsy, obstructive sleep apnoea, restless leg syndrome, periodic limb movements, and rapid-eye movement sleep behavioural disorder.^2^^,^^3^ Sleep disturbance, especially insomnia, is a common symptom of dementia and mild cognitive impairment (MCI), although as for people without dementia, factors such as pain, low mood, or a combination of causes may also disrupt sleep in this population.^3^ Some studies suggest that sleep disturbance has a key role in the progression of dementia^4^^–^6 or is associated with worsening symptoms.^7^

In a meta-analysis of 11 studies to explore the prevalence of sleep disturbance in people living with dementia (PLwD), 26% of the pooled population experienced sleep disturbance symptoms and 19% had clinically significant cases of sleep disturbance.^8^ Another review of 55 studies estimated a 38% prevalence of sleep disturbance among PLwD who were living in nursing homes based on symptoms, and 20% based on clinical measures.^9^ One literature review reported a 60% prevalence of any sleep disturbance among the MCI population, although studies exploring the prevalence of sleep disturbance among people with MCI are comparatively few.^10^^–^12

In primary care, the assessment and management of sleep disturbance among PLwD or MCI is complex because of a lack of clear diagnostic criteria and this population’s characteristics, which typically comprises older people living with multiple comorbidities, and polypharmacy.^13^ Although preliminary evidence suggests that non-pharmacological interventions are effective,^14^^–^16 these approaches are often not available, are not used or trusted by clinicians, and are not embedded within usual primary care practice.^17^

**Table:** How this fits in

Existing literature reports that the management of sleep disturbance among people living with dementia or mild cognitive impairment is a challenging problem. This realist review indicates why, how, and in which circumstances primary care is hindered when managing sleep disturbance in this population. Medication is often considered the primary management option because of a range of complex factors, despite limited efficacy and problematic side effects. Alternative management techniques, including evidence-based non-pharmacological strategies tailored to the individual and their carer/s, may improve sleep disturbance within this population.

The treatment of sleep disturbance among PLwD or MCI is a challenging or ‘wicked’ problem.^18^ Wicked problems originate from social planning and are defined as problems difficult or impossible to solve because of incomplete, contradictory, or changing requirements that are hard to define, and where solutions to one problem likely generate another problem.^19^ The assessment and management of sleep disturbance in primary care may therefore benefit from being examined carefully by considering all potential solutions, contexts, and experiences. A realist approach was therefore used to explore how primary care clinicians assess, understand, and manage sleep disturbance for PLwD or MCI; if and why such initiatives work; and how people and their carers experience sleep disturbance and its treatment.

## Method

This realist review is part of a larger National Institute for Health and Care Research funded research project.^20^ The review protocol is registered with PROSPERO (CRD42022304679) and published elsewhere.^21^

The realist approach recognises that an intervention’s impact is heavily dependent on its context and that a complex array of contextual factors therefore needs to be examined. Compared with other structured review approaches, the realist approach to review allows a wider array of search terms and broader inclusion criteria to be used, allowing for a more thorough identification and comparison of potential contextual factors.^22^

The bibliographic database search strategy was developed in Ovid MEDLINE by an information specialist (one of the authors) using a combination of free-text terms and controlled vocabulary (see Supplementary Box S1). No date limit was applied. The MEDLINE search was translated for use in an appropriate selection of bibliographic databases in total comprising APA, PsycINFO, HMIC, CINAHL, and ASSIA. The final searches were conducted on 4 July 2022. Forward citation searching and a reference list search of the included studies was also conducted.

The retrieved documents were imported to an EndNote X9 file and were examined for inclusion and exclusion (Box 1) through title, abstract, and then full-text screening (by three authors). Of the articles, 15% were randomly cross-checked by two authors for consistency at the title, abstract, and full-text screening stage. The recommended rate for cross-checking in realist reviews is 10%–20%.^23^ Discrepancies were discussed and screening strategies agreed on. The included articles were imported into NVivo (version 1.6.1).

**Box 1.** Inclusion and exclusion criteria

**Criteria**	**Include**	**Exclude**
**Population**	PLwD or MCI, primary/community-based clinicians, and carers (family, friends, unpaid, informal, or paid carers)	Trauma cases
**Phenomenon of interest**	Assessment and management of sleep	End-of-life care (life expectancy <3 months)
**Context and setting**	Primary care centres, community in the widest sense of the word (that is, live at home, sheltered accommodation, care homes including residential and nursing homes, or supported/assisted living setting)	Hospital-based/secondary/tertiary care or interventions, hospice care, non-Organisation for Economic Co-operation and Development nations
**Evaluation**	Experience of sleep disturbance, diagnosis, assessment, management, approaches, or interventions (what, how, why, by whom, for whom, what extent), experience with drug prescriptions, or medication usage	Quantitative findings from randomised controlled trials of effectiveness studies
**Study design/type of document**	Original research (published articles, conference abstracts, books, and unpublished and grey literature), reviews, viewpoints, policy documents, websites of professional bodies, and any relevant clinical or non-clinical guideline	—
**Other**	—	Non-English language

*MCI = mild cognitive impairment. PLwD = people living with dementia.*

The content was initially coded by two authors into three broad areas:

- assessment and diagnosis;
- management; and
- patient and carer experience and influence.

These categories were then refined into subcategories. Links between the data in each subcategory were discussed and arranged in an initial web of causation (see Supplementary Figure S1) from which the same two authors began drafting initial context–mechanism–outcome configurations (CMOCs).

CMOCs are the way that causal statements are expressed in realist reviews. Briefly, they set out how something that functions as context, is linked to an outcome, via a causal process called a mechanism. In other words, in a realist review, something that functions as context, ‘triggers’ a mechanism, which in turn causes an outcome.^24^

The developed CMOCs were refined with thorough cross-checking and reference to the primary data by three authors. Regular and substantial input was given throughout the process by one of the authors who is an expert in realist review methodology. The review followed RAMESES standards (see Supplementary Table S1).^24^

## RESULTS

### Study characteristics

**Figure 1.**
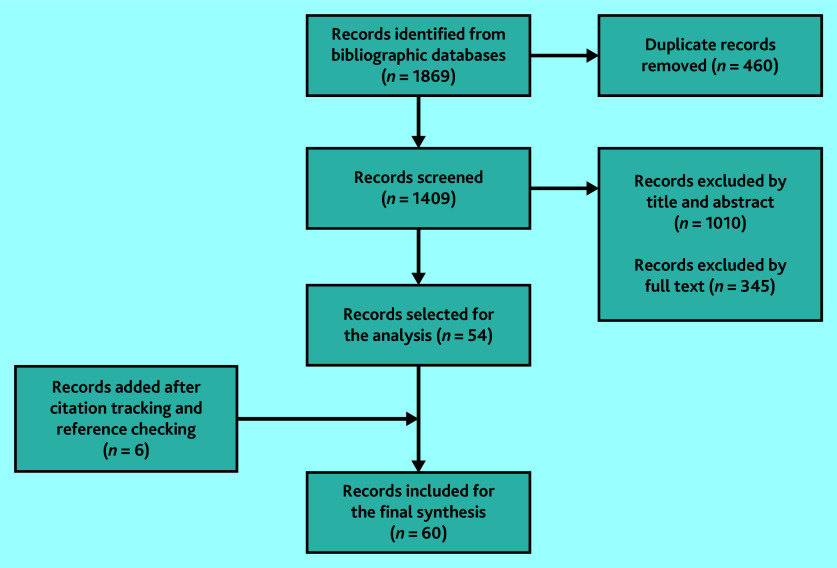
PRISMA flow diagram of screened documents.

The screening process (Figure 1) ended with 60 included records (see Supplementary Table S2). Most studies focused on the challenges of providing effective care. Reported challenges included problems in care homes,^25^^–^39 limited awareness or knowledge among clinicians,^39^^–^46 inappropriate use of medication,^16^^,^^36^^,^^44^^,^^47^^–^58 assessment and diagnosis difficulties,^32^^,^^39^^–^41^,^^43^^–^46^,^^59^^–^62 and negative experiences reported by PLwD or MCI and their carers.^31^^,^^34^^,^^41^^,^^57^^,^^63^^–^78 Few studies explored tailored care as a management strategy.^15^^,^^78^

Publication dates ranged between 1995 and 2022, with 50% of the included studies published within the past decade. Among the documents included, 36 were primary research, 15 were review articles, four were commentaries, and three book chapters. Additionally, one review protocol and one PhD thesis were incorporated, the latter of which had not been published in a peer-reviewed journal at the time of this review. Approximately one-third of the primary studies employed qualitative methodologies. Additionally, qualitative data and information were incorporated from the discussion and conclusion sections of the included studies.

In terms of geographical origin, most of the included articles were from the US (25 articles), followed by the UK (12 articles). Australia and Belgium each accounted for five documents, while Germany and Norway contributed three documents each. Canada, Austria, Ireland, Japan, the Netherlands, and New Zealand were each represented by one document, and one document was developed through international collaboration (UK and Canada).

### Context–mechanism–outcome configurations

In total, 19 CMOCs (see Supplementary Table S3) were developed, which are presented under four main themes:

- barriers to detection of sleep disturbance;
- long-term or inappropriate medication use;
- care homes’ role in the management of sleep disturbance; and
- positive role of informal carers.

#### Barriers to detection of sleep disturbance

**Figure 2.**
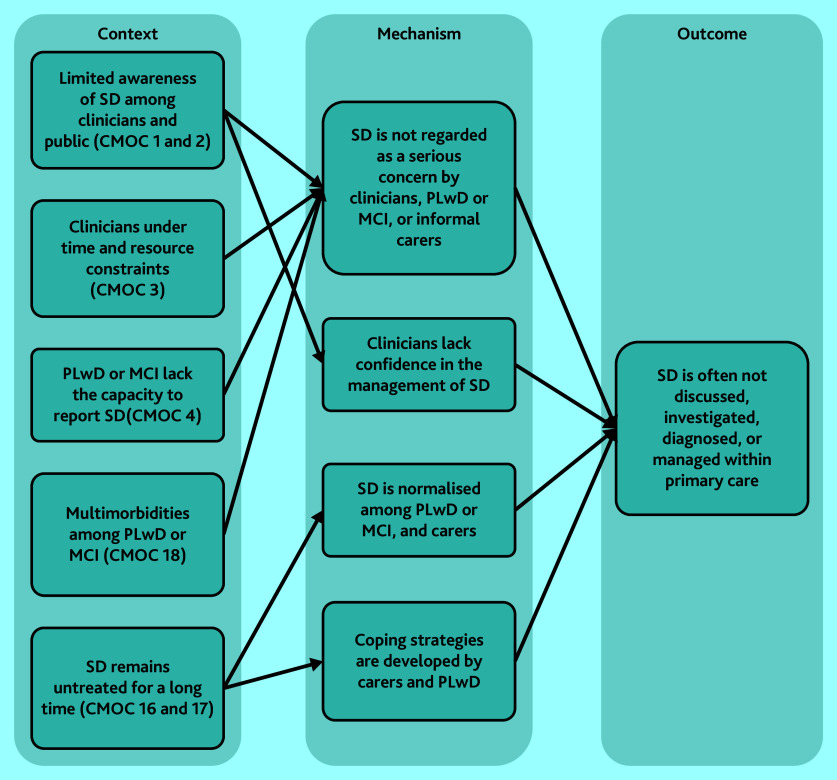
CMOCs that explain lack of sleep disturbance diagnosis among PLwD or MCI. CMOC = context–mechanism–outcome configuration; see Supplementary Table S3 for CMOC numbering. MCI = mild cognitive impairment. PLwD = people living with dementia. SD = sleep deprivation.

Multiple factors were found to limit discussion, investigation, detection, diagnoses, and management of sleep disturbance within primary care. Most CMOCs (1–4 and 16–18) explain the various reasons behind barriers or challenges and how they hinder appropriate intervention (Figure 2). These challenges result in sleep disturbance being unidentified or deprioritised as a health concern.^32^^,^^39^^–^41^,^^43^^–^46^,^^59^^–^62

There was limited awareness of sleep disturbance among clinicians, including its potential role in people’s health, possible assessment techniques, and non-pharmacological interventions, which contributed to sleep disturbance being unaddressed for many PLwD or MCI.^39^^–^46 Awareness of sleep disturbance among PLwD or MCI and their informal carers also appeared to be limited.^40^^,^^63^^,^^74^^,^^75^ The symptoms were sometimes normalised and coped with by the patient–carer dyad,^76^^,^^79^^,^^80^ or went unrecognised by the PLwD because of cognitive or communication impairments.^78^ This overall lack of awareness of sleep disturbance meant that discussions about sleep during primary care appointments did not frequently occur.

#### Long-term or inappropriate medication use

**Figure 3.**
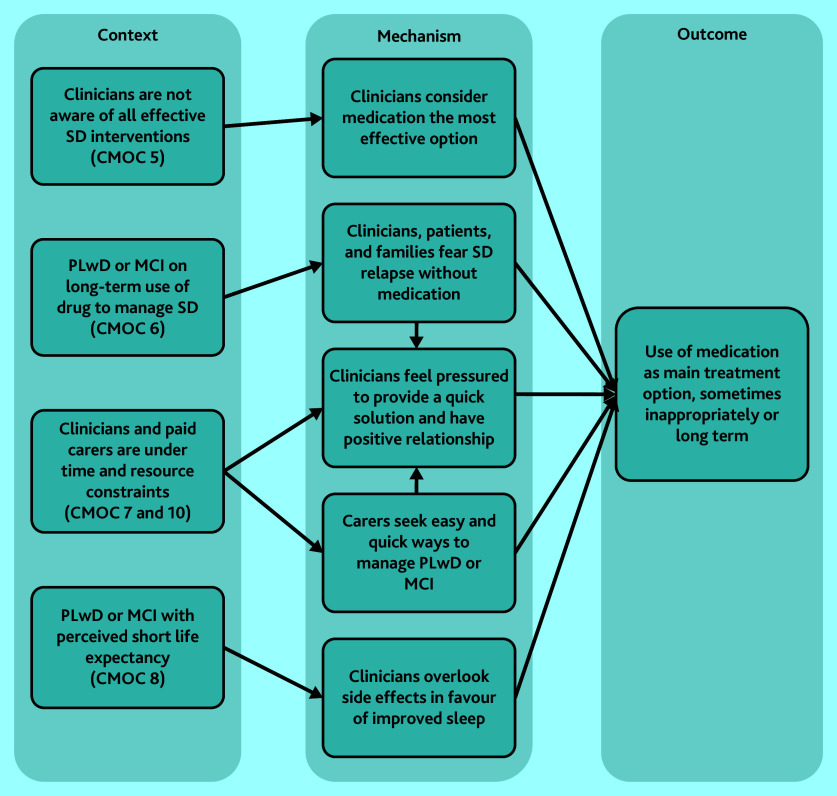
CMOCs that explain the causes for inappropriate or long-term use of medication. CMOC = context–mechanism–outcome configuration; see Supplementary Table S3 for CMOC numbering. MCI = mild cognitive impairment. PLwD = people living with dementia. SD = sleep deprivation.

In the instances where sleep disturbance is addressed in primary care, CMOCs 5–8 and 10 reveal how and why inappropriate prescription of sleep medication could manifest for PLwD or MCI (Figure 3). Clinicians’ limited awareness or lack of confidence in non-pharmaceutical interventions^15^^,^^16^^,^^54^ can result in medication being the preferred choice. Concerns about withdrawal effects if prescribed medications are ceased, from both clinicians and carers, also contributed to inappropriate long-term medication use.^47^^,^^50^ Carers relied on medication to relieve the stress of sleep deprivation,^25^^,^^81^ and prescribers reportedly felt compelled to prescribe medication to maintain positive relationships with their patients.^41^^,^^43^ The side effects of some medications were sometimes less of a concern for prescribers if the PLwD or MCI had a short life expectancy.^34^^,^^44^^,^^58^

#### Care homes’ role in the management of sleep disturbance

**Figure 4.**
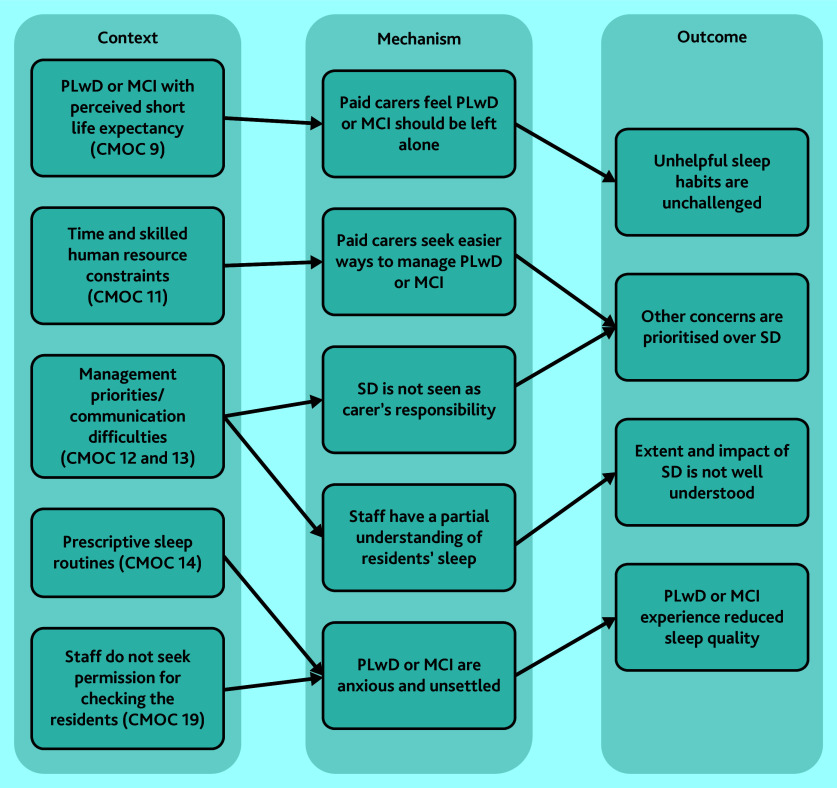
CMOCs explaining the role of care homes in the management of sleep disturbance. CMOC = context–mechanism–outcome configuration; see Supplementary Table S3 for CMOC numbering. MCI = mild cognitive impairment. PLwD = people living with dementia. SD = sleep deprivation.

Figure 4 demonstrates the relationships between CMOCs that explore care homes’ role in the management of sleep disturbance (CMOC 9, 11–14, and 19). Some concerns from the themes above, such as limited awareness of sleep disturbance, also apply to staff working in these settings.^28^^,^^29^^,^^31^^,^^32^^,^^37^ Care home staff were reluctant to implement sleep hygiene practices for PLwD or MCI for a range of reasons.^64^ As a result of environmental, resource, and policy-driven challenges, care homes reportedly imposed rigid routines for residents regardless of their individual circumstances.^33^^,^^38^ In some cases, night-time checks occurred without seeking consent, which was disruptive and anxiety-inducing for residents.^31^^,^^64^

#### Positive role of informal carers

**Figure 5.**
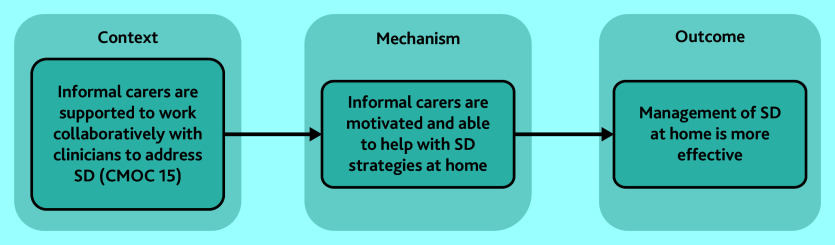
Role of informal carers in caring for a PLwD or MCI with sleep disturbance. CMOC = context–mechanism–outcome configuration; see Supplementary Table S3 for CMOC numbering. MCI = mild cognitive impairment. PLwD = people living with dementia. SD = sleep deprivation.

When informal carers were supported by health systems with information and active assistance (CMOC 15), they were able to follow recommended care plans for sleep management (Figure 5). Interventions for managing sleep disturbance among PLwD or MCI appeared to be dependent on carers, either informal or formal.^27^^,^^34^^,^^42^^,^^53^^,^^57^^,^^61^^,^^63^^,^^66^^,^^68^^–^70^,^^73^^,^^76^^,^^80^^,^^81^ Informal carers who were supported with information and guidance reported feeling more able and motivated to implement sleep management strategies at home. Carers also implemented coping strategies for handling sleep disturbance, which reportedly occurred independent of healthcare providers.^71^^,^^74^^,^^76^^,^^78^^,^^79^ However, the efficacy of these strategies was not explored in the literature.

## DISCUSSION

### Summary

The findings in this review highlight numerous challenges and barriers that impair effective assessment, diagnosis, and clinical management of sleep disturbance for PLwD or MCI. The diagnosis of sleep disturbance among this population is hindered by limited awareness of the issue among primary care clinicians and care home staff, compounded by time and resource constraints. PLwD or MCI who are unable to accurately report sleep disturbance can further challenge its identification. Long-term and inappropriate prescribing practices result from clinicians’ and patients’ beliefs that medication is the most effective and easiest ‘quick-fix’ management option. This is exacerbated by clinicians’ lack of confidence in the effectiveness or availability of evidence-based alternative, non-pharmacological interventions such as light therapy, cognitive behavioural therapy, physical activities, social relations with people, or even robopets, or sleep hygiene.^15^^,^^36^^,^^62^^,^^70^^,^^77^^,^^82^^,^^83^ In care homes, a lack of sufficiently trained staff and other resources, communication challenges, and conflicting organisational priorities resulted in rigid routines that have a negative impact on residents’ sleep. For those living in their own homes, informal carers play a significant role in the implementation of sleep management interventions.

### Strengths and limitations

This realist review was a detailed account of current literature on this topic. The review was led by one author who is a leading expert in realist review, and input was received throughout from those with expertise in the subject area. Arguably, the breadth of the inclusion criteria was also a limitation, as the large number of articles included from outside the UK made it difficult to ascertain whether the findings are wholly relevant to UK health provision. For example, there were some discrepancies between countries when reporting prescribing practices.^47^^,^^48^^,^^52^ However, many identified themes, such as low levels of awareness of sleep disturbance and the use of medication as the primary treatment method, were common across countries and healthcare systems.

Although including articles without a limit on date of publication allowed for a more comprehensive search, the authors of the current study cannot be sure that the findings detailed are entirely contemporary to current practice. Attitudes towards certain practices, such as the prescription of antipsychotic medications to treat sleep disturbance, have changed in recent years, with the UK Government pledging to reduce the prescription of antipsychotic medications for PLwD by two-thirds.^84^ Earlier UK-based research may not reflect this policy initiative and inappropriate use of this medication may no longer be contemporaneous with UK practice. However, there is evidence to suggest that although GPs are now aware of the negative consequences of sleep medication, there is a variable attitude to their use because of a perceived lack of alternatives and time constraints.^85^ Further, although many articles included detailed insight into sleep management practices in care homes, few studies explored current practices stemming from within GP surgeries and also few primary care professionals, excluding care home staff, were included as participants within the reviewed studies. More work is therefore required to accurately determine clinicians’ perspectives, and how current practice within primary care operates outside care homes, including general practice. A detailed exploration of current practice in the UK, including GP surgeries, is currently being conducted as the second phase of the TIMES project.

### Comparison with existing literature

Research exploring the general management of sleep disturbance in primary care identifies similar concerns regarding the inappropriate prescription of sleep medication and underutilisation of more tailored non-pharmacological solutions.^86^^–^88 Although practitioners may perceive a pressure to prescribe, this practice may actually deter patients with sleep disturbance from seeking further help.^89^ The use of sleep medication for PLwD or MCI can cause unintended consequences such as the progression of dementia, falls, and loss of functional abilities.^13^^,^^90^ Management of comorbidities, because of issues such as polypharmacy or use of medications for >1 condition, may further hinder deprescribing for different conditions.^91^ Like the findings in the current study, other studies indicate that when GPs work under time pressures they are prone to suboptimal prescribing practices.^91^^,^^92^ Resource pressures in care homes, such as understaffing and poor communication, was also linked to long-term medication use to manage sleep disturbance.^93^^,^^94^

### Implications for research and practice

The current review identified significant gaps within the literature. Further research is therefore needed to explore how sleep disturbance is assessed and managed for people attending GP surgeries and living in their own homes. Little information was available on how sleep disturbance is managed among people with MCI, and the literature did not readily distinguish between people with a dementia or MCI diagnosis. Future research could investigate sleep disturbance in the MCI population and focus on whether approaches do or should differ between PLwD and those with MCI. Finally, although much of the literature focused on the barriers to effective sleep management, few explored current and effective community-based sleep management practices. The authors of the current study would recommend that the perspectives of those implementing any sleep disturbance interventions, such as clinicians or, more likely, carers, are explored before any intervention is developed and more widely implemented. An understanding of carer perspectives is key to developing any effective sleep management tool within primary care.

The deprioritisation of sleep disturbance and lack of confidence in effective assessment and management strategies highlight the need to increase awareness of this area. This awareness should include instilling confidence in evidence-based non-pharmacological interventions, such as light therapy, cognitive behavioural therapy, physical activities, social engagement, and sleep hygiene.^15^^,^^36^^,^^62^^,^^70^^,^^77^^,^^82^ Integrating these components as part of a tailored approach to care may enable more effective interventions for sleep disturbance that are sensitive to the person’s unique circumstances and goals. It is essential, however, that any proposal for a new approach to care is implemented with due consideration for the time and resource constraints faced by healthcare and social service providers. The authors of the current study believe that patient care should include timely assessments of PLwD or MCI for possible sleep disturbances, a holistic approach to addressing sleep issues alongside various health conditions in this population, and the provision of continuous and context-specific care.

For PLwD or MCI with sleep disturbance living in care homes, a flexible and context-sensitive approach to managing sleep disturbance may help mitigate some of the negative experiences reported by care home residents at night. This might include adjusting sleep routines, limiting the number of night-time checks, and interventions relevant to residents’ preferences. Night-time care in care homes should be considered no less important than care during the day, and support and training for night staff to recognise, manage, and communicate sleep disturbance should be readily available.

PLwD or MCI, informal carers, and family members may also benefit from a greater awareness and understanding of the impact of poor sleep, good sleep hygiene practices, and advice on the potential side effects of sleep medication, including potentially severe adverse events.^13^ This could enable some elements of sleep disturbance to be effectively self-managed and alter the expectation or concern that doctors will prescribe as a first port of call. Notably, the current assessment and management of sleep disturbance for PLwD or MCI living at home is often dependent on the input of informal carers and families. Informal carers often experience high levels of burden associated with caring for a PLwD.^79^^,^^95^^,^^96^ Any intervention package involving informal carers should therefore be formulated collaboratively and with consideration as to how any proposed intervention can be feasibly implemented given their capabilities and available resources.
